# Supplementary material for: A Likelihood Approach for Real-Time Calibration of Stochastic Compartmental Epidemic Models
Source: PLoS Comput Biol. 2017 Jan 17;13(1):e1005257. doi: 10.1371/journal.pcbi.1005257 (PMC5240920; doi:10.1371/journal.pcbi.1005257)
Supplement: S1 File — (TAR.GZ) [file pcbi.1005257.s014.tar.gz › HSPH_Online-SI-Revision/EnKF07/n10-mild/n10-mild_EnKF_table0.pdf]

1	R0	{1.25046, 6.80278}	{2.66792, 7.45505}	{{2.01106, 3.41377}, {3., 10.7083}}	{118.81, 53.4282}	inf	157.	911.164	{350.191, 2052.39}	486.272	0.640976	newly	174.	1357.89	{292., 5705.}	686.695	0.574025	Reff	1.06127	0.863226	{0.314245, 2.13169}	57.269	ar	3516.	18600.	{16159., 24735.}	429.011
2	R0	{1.32013, 4.45847}	{2.2293, 7.22633}	{{0.627906, 0.23762}, {3., 11.705}}	{100.176, 135.745}	inf	321.	630.449	{64.4516, 814.14}	172.163	0.425914	newly	488.	741.77	{42., 1923.}	152.416	0.624868	Reff	0.980989	0.489454	{0.0655861, 0.835245}	59.4742	ar	4420.	17479.	{15790., 20256.}	295.452
3	R0	{1.31785, 5.36796}	{2.14301, 9.51079}	{{0.335054, 5.55616}, {3., 14.8974}}	{97.9599, 77.1771}	inf	363.	373.047	{65.2751, 309.649}	70.1945	0.322716	newly	488.	270.97	{28., 324.}	77.7643	0.605742	Reff	1.00407	0.406423	{0.0693008, 0.643171}	61.3569	ar	4942.	17519.	{16635., 18362.}	254.492
4	R0	{1.38801, 6.16031}	{2.58398, 10.722}	{{1.78919, 3.56628}, {3., 16.0978}}	{86.1643, 103.34}	inf	457.	468.025	{62.3772, 429.555}	85.7044	0.375416	newly	527.	301.21	{33., 695.}	77.4213	0.605723	Reff	1.01047	0.508737	{0.169398, 1.04362}	57.9279	ar	4790.	16698.9	{15591., 18021.}	248.619
5	R0	{1.27955, 11.57}	{2.79383, 10.0112}	{{1.87218, 4.35104}, {3., 14.4244}}	{118.345, 41.206}	inf	179.	1329.16	{1013.18, 1013.18}	642.548	0.80062	newly	120.	969.94	{423., 1166.}	708.533	0.797745	Reff	1.07866	0.636449	{0.216564, 1.5945}	58.9543	ar	3460.	18852.	{16762., 24199.}	444.855
6	R0	{1.22184, 4.34395}	{2.39599, 5.18927}	{{1.44738, 2.29343}, {3., 9.85318}}	{96.0972, 75.4414}	inf	231.	414.181	{24.2527, 1110.6}	139.862	0.425513	newly	353.	839.82	{22., 4353.}	222.601	0.610482	Reff	1.01352	0.657098	{0.0510057, 1.44283}	56.0668	ar	3524.	18817.5	{16760., 25089.}	433.982
7	R0	{1.23856, 4.59068}	{2.33344, 6.70511}	{{0.662125, 1.75442}, {3., 9.51477}}	{112.591, 81.8293}	inf	200.	631.997	{67.881, 1585.82}	267.013	0.422322	newly	288.	753.42	{44., 1747.}	231.188	0.494735	Reff	0.970659	0.61192	{0.107123, 0.993704}	57.1559	ar	3855.	17910.4	{15732., 22819.}	364.601
8	R0	{1.34531, 10.6689}	{2.46924, 4.15113}	{{1.20914, 2.39742}, {3., 5.85901}}	{96.6263, 61.0913}	inf	360.	1133.66	{491.854, 2879.9}	227.246	0.423054	newly	242.	1450.04	{360., 3890.}	509.636	0.617629	Reff	1.0562	0.528971	{0.111182, 1.09994}	62.5675	ar	4852.	19083.9	{16333., 24437.}	293.321
9	R0	{1.31775, 5.19226}	{2.82008, 12.4831}	{{2.30086, 6.79842}, {3., 16.4703}}	{114.007, 140.418}	inf	400.	435.977	{275.171, 275.171}	69.5522	0.208169	newly	527.	224.38	{92., 262.}	76.5844	0.552608	Reff	1.00689	0.552872	{0.294075, 0.885696}	51.3157	ar	4708.	16705.8	{15616., 17646.}	254.839
10	R0	{1.25916, 6.89207}	{2.68984, 6.58419}	{{2.30214, 3.74671}, {3., 15.1898}}	{116.147, 47.8092}	inf	255.	383.809	{142.326, 488.317}	70.6947	0.228188	newly	246.	537.01	{108., 835.}	149.996	0.333132	Reff	0.978871	0.692935	{0.239665, 2.02314}	70.3267	ar	3823.	17389.8	{15810., 21600.}	354.873
11	R0	{1.20377, 5.46566}	{2.49879, 3.83226}	{{1.65289, 3.50936}, {3., 4.40688}}	{107.58, 29.8847}	inf	165.	428.026	{49.5968, 690.891}	185.221	0.312657	newly	221.	627.58	{51., 867.}	217.195	0.366697	Reff	0.992749	0.588458	{0.155587, 0.866906}	61.4937	ar	3114.	18692.	{16632., 25701.}	500.257
12	R0	{1.27878, 2.55804}	{2.18781, 9.0942}	{{0.312834, 5.42247}, {3., 13.6989}}	{103.486, 255.515}	inf	209.	362.638	{34.6054, 484.181}	110.155	0.390614	newly	522.	267.8	{27., 306.}	76.6245	0.657443	Reff	0.948983	0.451297	{0.0691438, 0.664422}	53.1133	ar	3930.	17481.5	{16680., 17739.}	344.821
13	R0	{1.41227, 5.45696}	{2.55151, 3.72343}	{{1.96861, 2.39554}, {3., 5.87269}}	{90.5309, 35.8992}	inf	402.	995.433	{488.165, 2998.05}	153.404	0.264467	newly	505.	1214.23	{388., 2680.}	154.604	0.273931	Reff	1.01556	0.49501	{0.127856, 1.3095}	65.6593	ar	4822.	18369.4	{15761., 23979.}	280.949
14	R0	{1.32917, 7.27593}	{2.58168, 4.27731}	{{1.34323, 2.91729}, {3., 6.92588}}	{102.054, 41.2129}	inf	374.	599.518	{78.8516, 1507.01}	91.1817	0.281585	newly	351.	874.92	{66., 1729.}	180.644	0.372627	Reff	0.994485	0.70119	{0.132712, 1.35599}	59.559	ar	4758.	17874.2	{15844., 21422.}	275.665
15	R0	{1.31629, 9.97481}	{2.60359, 2.29163}	{{1.81498, 0.683669}, {3., 3.2597}}	{97.7975, 77.0258}	inf	325.	396.225	{45.4294, 481.88}	89.3373	0.382125	newly	238.	685.13	{52., 1362.}	223.038	0.428085	Reff	1.102	0.500473	{0.120784, 1.01482}	64.1726	ar	4543.	16965.4	{15813., 18789.}	273.441
16	R0	{1.36735, 5.74166}	{2.74858, 7.99777}	{{2.30499, 3.16626}, {3., 10.1863}}	{101.015, 55.4429}	inf	345.	502.085	{80.4919, 291.383}	103.119	0.261307	newly	435.	419.63	{90., 366.}	104.816	0.46222	Reff	1.05464	0.625148	{0.290396, 1.09679}	52.0251	ar	4516.	17398.4	{15715., 18782.}	285.26
17	R0	{1.27524, 13.2396}	{2.6553, 3.76093}	{{1.9032, 2.99172}, {3., 5.08406}}	{108.22, 71.5933}	inf	189.	909.436	{418.216, 2765.6}	383.113	0.482662	newly	111.	1211.4	{344., 3564.}	993.009	0.802188	Reff	1.19643	0.572541	{0.142574, 1.32804}	63.0153	ar	3731.	18654.6	{16235., 25974.}	399.989
18	R0	{1.21884, 7.71545}	{2.73698, 9.46506}	{{2.32911, 6.86633}, {3., 12.084}}	{124.608, 33.6822}	inf	231.	511.411	{44.936, 337.59}	183.709	0.395118	newly	192.	381.34	{32., 313.}	178.24	0.480854	Reff	1.02078	0.577002	{0.249441, 1.33027}	66.6698	ar	3080.	17352.9	{15423., 23511.}	463.405
19	R0	{1.34879, 6.71464}	{2.72933, 11.3164}	{{2.28981, 6.51986}, {3., 13.8153}}	{102.354, 70.5061}	inf	378.	409.968	{128.255, 285.367}	83.2558	0.285278	newly	370.	247.88	{52., 225.}	87.2486	0.503147	Reff	0.926888	0.653555	{0.218835, 1.16915}	52.3357	ar	4585.	17355.2	{15800., 17910.}	278.52
20	R0	{1.37284, 5.75036}	{2.31414, 6.71872}	{{0.689563, 1.03277}, {3., 9.27836}}	{95.404, 64.3987}	inf	291.	707.298	{58.0887, 1534.05}	215.33	0.45041	newly	356.	727.16	{34., 1728.}	194.18	0.562068	Reff	1.04981	0.568765	{0.103641, 0.912353}	60.7464	ar	4750.	17768.5	{15576., 23223.}	274.073
21	R0	{1.29938, 5.36419}	{2.25659, 6.76844}	{{0.639796, 0.310429}, {3., 11.8466}}	{102.958, 108.896}	inf	347.	790.719	{77.092, 1515.}	200.759	0.446398	newly	456.	814.94	{42., 1610.}	163.373	0.576166	Reff	0.968298	0.477426	{0.0797661, 0.817694}	56.9435	ar	4337.	17634.6	{15804., 21109.}	306.608
22	R0	{1.31882, 7.47626}	{2.63516, 3.86318}	{{1.6813, 3.06554}, {3., 4.84097}}	{101.435, 48.3273}	inf	278.	705.029	{67.019, 1797.95}	182.191	0.424436	newly	256.	1009.29	{70., 2627.}	320.16	0.520852	Reff	1.04292	0.606861	{0.164242, 1.34879}	60.2295	ar	3977.	18371.5	{15943., 22100.}	361.945
23	R0	{1.26215, 3.46375}	{2.57499, 6.40692}	{{1.65855, 3.58631}, {3., 9.01056}}	{104.016, 84.9707}	inf	212.	507.169	{104.377, 743.013}	155.285	0.239859	newly	394.	597.37	{77., 539.}	128.617	0.373233	Reff	0.984098	0.672513	{0.254503, 0.981718}	52.7476	ar	3607.	18511.	{16273., 25608.}	413.197
24	R0	{1.16712, 4.19464}	{2.30505, 2.31689}	{{0.994139, 0.396844}, {3., 8.41169}}	{106.504, 80.0407}	inf	126.	253.305	{59.3022, 204.496}	118.282	0.26025	newly	188.	280.15	{82., 425.}	74.7287	0.234398	Reff	0.946418	0.329799	{0.0286674, 0.665238}	65.3521	ar	3000.	16880.8	{15769., 18643.}	462.692
25	R0	{1.22751, 3.34517}	{2.41729, 5.26935}	{{1.45982, 2.18749}, {3., 9.80917}}	{96.9262, 94.365}	inf	170.	384.063	{18.8619, 1080.44}	174.862	0.437457	newly	366.	721.8	{20., 4198.}	193.224	0.638636	Reff	1.07714	0.624019	{0.0625032, 1.45266}	58.897	ar	3086.	18749.6	{16815., 25102.}	507.569
26	R0	{1.18747, 5.88083}	{2.65814, 5.2922}	{{1.97318, 3.51828}, {3., 7.15779}}	{127.63, 30.968}	inf	219.	644.465	{48.6689, 601.003}	224.245	0.296267	newly	259.	631.1	{38., 1225.}	192.958	0.387491	Reff	0.891909	0.548741	{0.018422, 1.18806}	65.709	ar	3614.	17624.4	{15224., 24243.}	387.67
27	R0	{1.29023, 4.76202}	{2.30701, 2.07986}	{{0.911551, 0.387041}, {3., 3.45274}}	{95.6194, 56.3239}	inf	314.	413.041	{15.286, 757.268}	104.798	0.456285	newly	412.	608.44	{31., 828.}	109.408	0.445344	Reff	0.948061	0.419561	{0.105174, 0.648361}	61.927	ar	4245.	16629.8	{15337., 18101.}	291.749
28	R0	{1.2258, 12.9056}	{2.67834, 10.2896}	{{1.71333, 1.74909}, {3., 19.6028}}	{118.497, 56.4068}	inf	204.	1003.91	{82.9021, 1209.95}	413.06	0.629409	newly	111.	876.01	{31., 1128.}	714.91	0.780564	Reff	0.977943	0.536986	{0.314557, 1.04022}	58.0617	ar	3639.	14760.3	{12349., 21601.}	305.613
29	R0	{1.34702, 6.29049}	{2.62379, 1.98553}	{{1.78314, 0.603234}, {3., 3.2815}}	{94.7845, 68.436}	inf	319.	302.682	{82.2596, 444.78}	57.7334	0.283481	newly	360.	681.41	{126., 1463.}	135.014	0.325991	Reff	0.99693	0.530619	{0.0391749, 0.946513}	56.6325	ar	4560.	16257.	{15406., 17460.}	256.512
30	R0	{1.37931, 6.78741}	{2.20003, 7.01824}	{{0.957795, 3.46623}, {3., 11.1681}}	{79.9451, 51.3537}	inf	364.	348.898	{63.4159, 443.672}	58.9531	0.306044	newly	361.	414.45	{50., 500.}	100.008	0.416958	Reff	0.975724	0.560752	{0.142031, 1.0078}	57.7691	ar	4667.	16728.3	{15529., 18622.}	258.438
31	R0	{1.16062, 4.47529}	{2.4369, 5.4171}	{{1.45333, 2.20337}, {3., 9.84064}}	{109.965, 76.7285}	inf	237.	347.772	{18.9218, 1058.6}	123.265	0.490893	newly	360.	720.47	{17., 3236.}	199.753	0.673893	Reff	0.985599	0.625352	{0.0529708, 1.44632}	58.0773	ar	3037.	18671.9	{16796., 24939.}	514.815
32	R0	{1.37647, 18.7122}	{2.62595, 2.86036}	{{1.81019, 1.87316}, {3., 3.33965}}	{90.7743, 84.7139}	inf	357.	317.395	{132.51, 971.084}	92.5864	0.435882	newly	145.	886.79	{126., 4050.}	520.421	0.311549	Reff	1.02478	0.522016	{0.0889323, 1.81066}	74.6228	ar	4824.	17116.2	{15452., 24812.}	254.812
33	R0	{1.35084, 6.08841}	{2.57831, 2.01557}	{{1.76776, 0.741202}, {3., 3.27412}}	{90.8675, 66.895}	inf	485.	449.051	{152.588, 428.796}	71.8153	0.299198	newly	533.	740.04	{209., 1193.}	86.0188	0.258394	Reff	0.972335	0.511605	{0.1697, 0.819834}	51.2202	ar	4759.	16444.	{15603., 17159.}	245.535
34	R0	{1.36912, 6.316}	{2.63113, 2.18094}	{{1.79711, 0.773747}, {3., 3.2693}}	{92.1767, 65.4695}	inf	294.	281.457	{52.4382, 476.561}	60.3632	0.319026	newly	335.	632.54	{90., 1618.}	143.54	0.363936	Reff	1.06983	0.499756	{0.126885, 0.771787}	62.0421	ar	4537.	16280.6	{15370., 17450.}	258.842
35	R0	{1.35831, 7.09038}	{2.6518, 2.03098}	{{1.76243, 0.534268}, {3., 3.28115}}	{95.2282, 71.3558}	inf	375.	465.38	{104.957, 487.056}	91.8576	0.326558	newly	358.	927.7	{160., 1582.}	188.704	0.319208	Reff	0.949459	0.64563	{0.31328, 0.957753}	52.6668	ar	4667.	16824.4	{15547., 18635.}	260.496
36	R0	{1.28315, 5.89938}	{2.25116, 6.69227}	{{0.62309, 0.445863}, {3., 10.2139}}	{105.106, 79.9357}	inf	365.	738.902	{26.1854, 2233.12}	203.009	0.63207	newly	398.	842.43	{27., 3043.}	217.661	0.760822	Reff	0.92297	0.523755	{0.0549482, 0.87725}	57.0917	ar	4460.	17512.6	{15543., 22710.}	292.658
37	R0	{1.19303, 2.75113}	{2.1743, 9.56303}	{{0.388441, 5.61217}, {3., 14.1518}}	{112.796, 247.604}	inf	171.	361.592	{59.5145, 291.27}	143.829	0.342527	newly	357.	271.37	{30., 298.}	85.2353	0.551346	Reff	0.971365	0.403596	{0.0804926, 0.664888}	60.1406	ar	3142.	17603.4	{16681., 18182.}	460.262
38	R0	{1.36033, 7.8088}	{2.69461, 11.9854}	{{2.29487, 6.67129}, {3., 15.2772}}	{98.0851, 64.5574}	inf	377.	399.391	{236.596, 484.398}	66.3266	0.228451	newly	348.	217.66	{91., 261.}	67.7701	0.408977	Reff	1.02419	0.55658	{0.221958, 0.802284}	55.6918	ar	4941.	17019.7	{15731., 18048.}	244.458
39	R0	{1.27353, 3.70845}	{2.25852, 6.71073}	{{0.642869, 1.77634}, {3., 9.55883}}	{105.919, 108.034}	inf	255.	548.518	{79.8224, 430.501}	174.449	0.325961	newly	460.	628.45	{50., 471.}	150.176	0.587356	Reff	0.944322	0.491997	{0.0824455, 0.85377}	56.8064	ar	3725.	17291.1	{15625., 19506.}	364.19
40	R0	{1.19983, 12.1486}	{2.88633, 7.86409}	{{2.18806, 3.90895}, {3., 14.9284}}	{140.561, 50.1906}	inf	151.	79.2665	{51.7969, 107.422}	65.1555	0.405656	newly	79.	58.07	{26., 91.}	49.1013	0.264166	Reff	0.976302	0.359775	{0.0151585, 0.98257}	67.9077	ar	3619.	15922.3	{15435., 16902.}	339.963
41	R0	{1.20935, 2.67169}	{2.45739, 5.61896}	{{1.43494, 2.25836}, {3., 9.83991}}	{103.199, 124.768}	inf	236.	471.48	{30.4599, 1203.32}	181.257	0.515123	newly	573.	713.05	{31., 2471.}	155.373	0.797318	Reff	0.945228	0.569413	{0.0796704, 1.41157}	57.3978	ar	3429.	18558.7	{16696., 24530.}	441.227
42	R0	{1.23313, 4.74781}	{2.64615, 11.6327}	{{1.7392, 4.1503}, {3., 16.3461}}	{115.166, 152.51}	inf	192.	379.994	{48.137, 402.349}	132.675	0.325293	newly	278.	247.03	{27., 630.}	90.6295	0.497223	Reff	0.975036	0.624106	{0.0946857, 1.45606}	65.5441	ar	3835.	16910.6	{15298., 21749.}	340.955
43	R0	{1.26105, 2.39713}	{2.41541, 8.56576}	{{0.528854, 1.29254}, {3., 18.8186}}	{111.576, 307.83}	inf	151.	469.381	{77.8528, 637.049}	233.933	0.345469	newly	396.	877.71	{67., 1827.}	224.078	0.572898	Reff	0.95865	0.514241	{0.0536819, 0.798477}	62.0216	ar	3723.	17942.5	{16476., 19510.}	381.936
44	R0	{1.36948, 8.88006}	{2.52821, 10.9993}	{{1.81284, 1.62534}, {3., 16.7909}}	{84.6113, 85.8815}	inf	376.	385.28	{45.7766, 414.921}	89.17	0.415499	newly	309.	287.92	{33., 612.}	99.3657	0.550362	Reff	1.06984	0.524552	{0.160203, 0.894425}	62.3966	ar	4437.	16677.1	{15488., 17762.}	275.863
45	R0	{1.34882, 3.3791}	{2.47015, 5.28667}	{{1.99707, 4.0564}, {3., 7.44434}}	{85.5346, 58.4053}	inf	365.	253.358	{14.6147, 295.28}	91.4006	0.607891	newly	717.	294.41	{12., 572.}	81.2343	0.808204	Reff	1.04641	0.597803	{0.179475, 1.99687}	66.3886	ar	4422.	18355.1	{16864., 24872.}	315.364
46	R0	{1.23774, 4.34917}	{2.44306, 8.15822}	{{0.960184, 1.46245}, {3., 19.3942}}	{107.895, 163.114}	inf	261.	403.176	{69.0711, 658.783}	103.267	0.324675	newly	370.	1101.07	{75., 2102.}	295.765	0.575949	Reff	0.996752	0.676626	{0.15858, 1.62698}	56.5153	ar	3685.	18381.9	{16545., 24777.}	398.83
47	R0	{1.34327, 4.78811}	{2.46748, 8.34165}	{{0.871146, 1.39448}, {3., 19.2575}}	{97.1708, 156.07}	inf	281.	467.414	{44.2673, 829.837}	129.059	0.372158	newly	448.	1023.21	{24., 1641.}	239.109	0.673574	Reff	1.0953	0.604541	{0.0967022, 1.07609}	58.8289	ar	4517.	18021.1	{16473., 18692.}	298.961
48	R0	{1.24366, 4.25814}	{2.74634, 11.8306}	{{2.30612, 6.65196}, {3., 14.764}}	{120.827, 177.834}	inf	209.	369.411	{255.511, 277.492}	79.9832	0.149994	newly	310.	207.89	{97., 250.}	63.8613	0.346974	Reff	0.987217	0.576115	{0.252553, 0.866106}	53.4876	ar	3678.	17030.4	{15759., 18486.}	363.035
49	R0	{1.36017, 7.5415}	{2.64275, 2.73195}	{{1.99038, 2.1065}, {3., 3.41615}}	{102.111, 63.7745}	inf	190.	600.491	{32.2213, 845.825}	251.498	0.491667	newly	183.	1393.57	{47., 3199.}	690.519	0.687536	Reff	1.12513	0.803563	{0.109925, 1.99018}	60.1794	ar	4178.	17869.	{15515., 24829.}	327.692
50	R0	{1.25333, 6.63578}	{2.74548, 6.15118}	{{1.99719, 3.45642}, {3., 13.1234}}	{120.579, 33.4419}	inf	193.	487.28	{162.717, 978.252}	163.048	0.233368	newly	204.	899.83	{118., 3046.}	360.789	0.287563	Reff	0.945763	0.80069	{0.156647, 2.38955}	62.9514	ar	3401.	17259.3	{15310., 24129.}	407.478
